# Efficacy and safety of HIP1601 (dual delayed-release esomeprazole) 40 mg in erosive esophagitis compared to HGP1705 (delayed-release esomeprazole) 40 mg: a multicenter, randomized, double-blind, non-inferiority study

**DOI:** 10.1186/s12876-023-03087-6

**Published:** 2023-12-18

**Authors:** Hyun Lim, Jong Kyu Park, Hyunsoo Chung, Si Hyung Lee, Jae Myung Park, Jung Ho Park, Gwang Ha Kim, Sung Kwan Shin, Su Jin Hong, Kwang Jae Lee, Moo In Park, Hye-Kyung Jung, Hyun-Soo Kim, Jae Kyu Sung, Seong Woo Jeon, Suck Chei Choi, Jeong Seop Moon, Nayoung Kim, Jong-Jae Park, Sung Hee Hong, Na Young Kim, Hwoon-Yong Jung

**Affiliations:** 1 Department of Internal Medicine, University of Hallym College of Medicine, Hallym University Sacred Heart Hospital, Anyang, Republic of Korea; 2 Division of Gastroenterology, Department of Internal Medicine, University of Ulsan College of Medicine, Gangneung Asan Hospital, Gangneung, Republic of Korea; 3 Department of Internal Medicine and Liver Research Institute, Seoul National University College of Medicine, Seoul, Republic of Korea; 4 Department of Internal Medicine, Yeungnam University College of Medicine, Daegu, Republic of Korea; 5 Department of Internal Medicine, Seoul St. Mary’s Hospital, College of Medicine, The Catholic University of Korea, Seoul, Republic of Korea; 6 Department of Internal Medicine, Kangbuk Samsung Hospital, Sungkyunkwan University College of Medicine, Seoul, Republic of Korea; 7 Department of Internal Medicine, Biomedical Research Institute, Pusan National University School of Medicine, Pusan National University Hospital, Busan, Republic of Korea; 8 Division of Gastroenterology, Institute of Gastroenterology, Department of Internal Medicine, Yonsei University College of Medicine, Seoul, Republic of Korea; 9 Digestive Disease Center and Research Institute, Department of Internal Medicine, Soonchunhyang University College of Medicine, Bucheon, Republic of Korea; 10 Department of Gastroenterology, Ajou University School of Medicine, Suwon, Republic of Korea; 11 Department of Internal Medicine, Kosin University College of Medicine, Busan, Republic of Korea; 12 Department of Internal Medicine, Ewha Womans University School of Medicine, Seoul, Republic of Korea; 13 Department of internal medicine, Chonnam National University Hospital, Gwangju, Republic of Korea; 14 Department of Internal Medicine, Chungnam National University College of Medicine, Daejeon, Republic of Korea; 15 Department of Internal Medicine, School of Medicine, Kyungpook National University, Daegu, Republic of Korea; 16 Department of Gastroenterology, Digestive Disease Research Institute, Wonkwang University Hospital, Iksan, Republic of Korea; 17 Department of Internal Medicine, Inje University College of Medicine, Seoul, Republic of Korea; 18 Department of Internal Medicine, Seoul National University Bundang Hospital, Seongnam, Republic of Korea; 19 Division of Gastroenterology, Department of Internal Medicine, Korea University College of Medicine, Guro Hospital, Seoul, Republic of Korea; 20 Hanmi Pharmaceutical Co., Ltd, Seoul, Republic of Korea; 21 Department of Gastroenterology, University of Ulsan College of Medicine, Asan Medical Center, Seoul, Republic of Korea

**Keywords:** Proton pump inhibitor, HIP1601, Esomeprazole, Gastroesophageal reflux disease

## Abstract

**Background:**

Proton-pump inhibitors (PPIs) are the most effective drugs for treating acid-related disorders. However, once-daily dosing with conventional PPIs fail to fully control acid secretion over 24 h. This study aimed to compare the efficacy and safety of HIP1601 (dual delayed-release esomeprazole) and HGP1705 (delayed-release esomeprazole) in patients with erosive esophagitis (EE).

**Methods:**

We enrolled 213 patients with EE randomized in a 1:1 ratio to receive 40 mg HIP1601 (n = 107) or HGP1705 (n = 106) once daily for 4 or 8 weeks. The primary endpoint was the EE healing rate, confirmed by endoscopy up to week 8. GERD-related symptoms and treatment-emergent adverse events were compared between both groups.

**Results:**

By week 8, the estimated healing rates of EE were 97.8% and 96.8% in the HIP1601 and HGP1705 groups, respectively, with a 95% confidence interval of -4.7 to 7.2. After 4 or 8 weeks of treatment, the EE healing rate at week 4, complete resolution rate of symptoms, time to sustained resolution of symptoms, and number of rescue medications used were similar in both groups. The proportion of heartburn- and acid regurgitation-free nights by week 4 were higher in the HIP1601 group compared to the HGP1705 group, but the difference did not reach clinical significance (87.7% vs. 85.8%, *P* = 0.514, 87.5% vs. 85.8%, *P* = 0.774). The number of adverse events did not differ significantly between the two groups.

**Conclusions:**

The efficacy and safety of HIP1601 40 mg were comparable to those of HGP1705 40 mg for the treatment of EE and symptomatic improvement of GERD.

**Trial registration:**

NCT04080726 (https://classic.clinicaltrials.gov/ct2/show/NCT04080726), registration date: 25/10/2018.

**Supplementary Information:**

The online version contains supplementary material available at 10.1186/s12876-023-03087-6.

## Background

Gastroesophageal reflux disease (GERD) is a chronic disorder caused by the reflux of gastric contents, resulting in symptoms such as heartburn and acid regurgitation as well as potential complications such as esophagitis and Barrett’s esophagus [1]. Although GERD does not directly cause patient mortality, it can significantly affect the quality of life. Numerous studies have shown that patients with GERD experience a lower quality of life than healthy individuals [2, 3]. The quality of life of patients with GERD is similar to that of patients with diabetes, malignancy, and coronary artery disease and lower than that of patients with peptic ulcers, hypertension, heart failure, and menopause [4]. Consequently, an effective treatment is necessary to alleviate GERD symptoms.

Proton pump inhibitors (PPIs) are the most commonly prescribed class of medications to treat GERD. They function by inhibiting the secretion of hydrogen ions through the inhibition of H+/K+-ATPase (a proton pump) located on the secretory surface of gastric parietal cells, resulting in a potent inhibition of gastric acid secretion and a sustained increase of intragastric pH. Previous studies have consistently demonstrated the superiority of PPIs over other acid blockers and placebo in controlling GERD symptoms and preventing complications such as esophagitis, esophageal stricture, and Barrett’s esophagus [5, 6]. However, PPIs have a short half-life (less than 2 h) and only inhibit around 70% of proton pumps [7]. When symptom control is the primary goal, a significant number of patients with GERD do not respond adequately to conventional, once-daily PPI therapy. Gastric acid may not always play a crucial role in patients with poorly controlled GERD symptoms. Hence, there is a requirement for better acid control through more effective PPIs or improved PPI delivery.

HIP1601 is a newly developed esomeprazole formulation with dual delayed-release emission. It comprises a mixture of two types of enteric-coated granules designed to dissolve at different pH levels. Compared to conventional delayed-release PPIs, HIP1601 has an extended duration of therapeutic plasma drug concentration and is expected to improve the efficacy of GERD treatment [8]. However, the clinical data on the efficacy and safety of HIP1601 are limited.

This study aimed to demonstrate that the clinical efficacy and safety of HIP1601 40 mg are non-inferior to those of HGP1705 (delayed-release esomeprazole, Nexium®) 40 mg in patients with GERD.

## Materials and methods

### Study design

This study was designed as a multicenter, double-blind, randomized controlled, non-inferiority trial involving patients with erosive esophagitis (EE). Patients with EE were randomly assigned to receive either HIP1601 40 mg or HGP1705 40 mg for 4 or 8 weeks. Complete EE healing rates, GERD-related symptoms, and treatment-emergent adverse events (TEAE) were investigated and compared between both drugs. This study was conducted at 20 hospitals in Republic of Korea between October 2018 and November 2019. The study protocol was approved by the ethics committees of all participating hospitals, and written informed consent was obtained from each patient before enrollment (Asan Medical Center Institutional Review Board [A2018-1391], CMC Institutional Review Board [KC18MDDT0645], GangNeung Asan Hsopital Institutional Review Board [GNAH 2018-09-012], Kangbuk Samsung Hospital Institutional Review Board [KBSMC 2018-08-040], Korea University Guro Hospital Institutional Review Board [2018GR0399], Kosin University Gospel Hospital Institutional Review Board [KUGH 2018-08-029], Pusan National University Hospital Institutional Review Board [D-1809-003-081], Seoul National University Hospital Institutional Review Board [H-1808-183-970], Severance Hospital Institutional Review Board [4-2018-1229], Soonchunhyang University Hospital Bucheon Institutional Review Board [SCHBC 2018-09-005], Ajou University Hospital Institutional Review Board [AJIRB-MED-CT3-18-327], Yeungnam University Hospital Institutional Review Board [2018-08-045], Wonkwang University Hospital Institutional Review Board [WKUH 2018-09-008], Ewha womans university medical center Institutional Review Board [EUMC 2018-09-004], Inje University Seoul Paik Hospital Institutional Review Board [PAIK 2018-09-003], Chonnam National University Hospital Institutional Review Board [CNUH-2018-237], Chungnam National University Hospital Institutional Review Board [CNUH-2018-063], Kyungpook National University Chilgok Hospital Institutional Review Board [KNUCH 2018-08-035], Hallym University Sacred Heart Hospital Institutional Review Board [HALLYM 2018-08-033] & Seoul National University Bundang Hospital Institutional Review Board [B-1904-532-405]). This trial has been registered in Clinical Trials. (NCT04080726, registration date: 25/10/2018, https://clinicaltrials.gov/study/NCT04080726).

### Study subject

Male and female patients aged 19–75 years were eligible for inclusion if they had endoscopically confirmed EE based on the Los Angeles (LA) Classification (grades A–D). Additionally, patients had to experience symptoms of acid regurgitation and/or heartburn 7 days prior to the screening visit.

The exclusion criteria for the study were as follows: (1) patients who had taken PPI and histamine receptor 2 blocking agent within 2 weeks before the screening endoscopy, (2) patients with active gastric or duodenal ulcer, gastrointestinal bleeding, primary esophageal motility disorders, Zollinger-Ellison syndrome, inflammatory bowel disease, malignancy, and endoscopic Barrett’s esophagus more than 3 cm or esophageal dysplasia, (3) patients who had undergone previous major abdominal surgery, and (4) patients with abnormal laboratory findings at the screening (MDRD eGFR ≤ 59 mL/min/1.73 m^2^ or serum creatinine > 2.0 mg/dL; serum levels of alanine aminotransferase or aspartate aminotransferase > 3 upper limit of normal). Pregnant or lactating women and those requiring hospitalization for surgery were excluded. The patients were not allowed to use any concomitant medications that could affect the evaluation of efficacy, including PPIs, histamine receptor 2 blocking agents, antidepressants, antipsychotics, or antianxiety drugs. Further exclusion criteria can be found at ClinicalTrials.gov (number NCT04080726).

### Study protocol

**Fig. 1:**
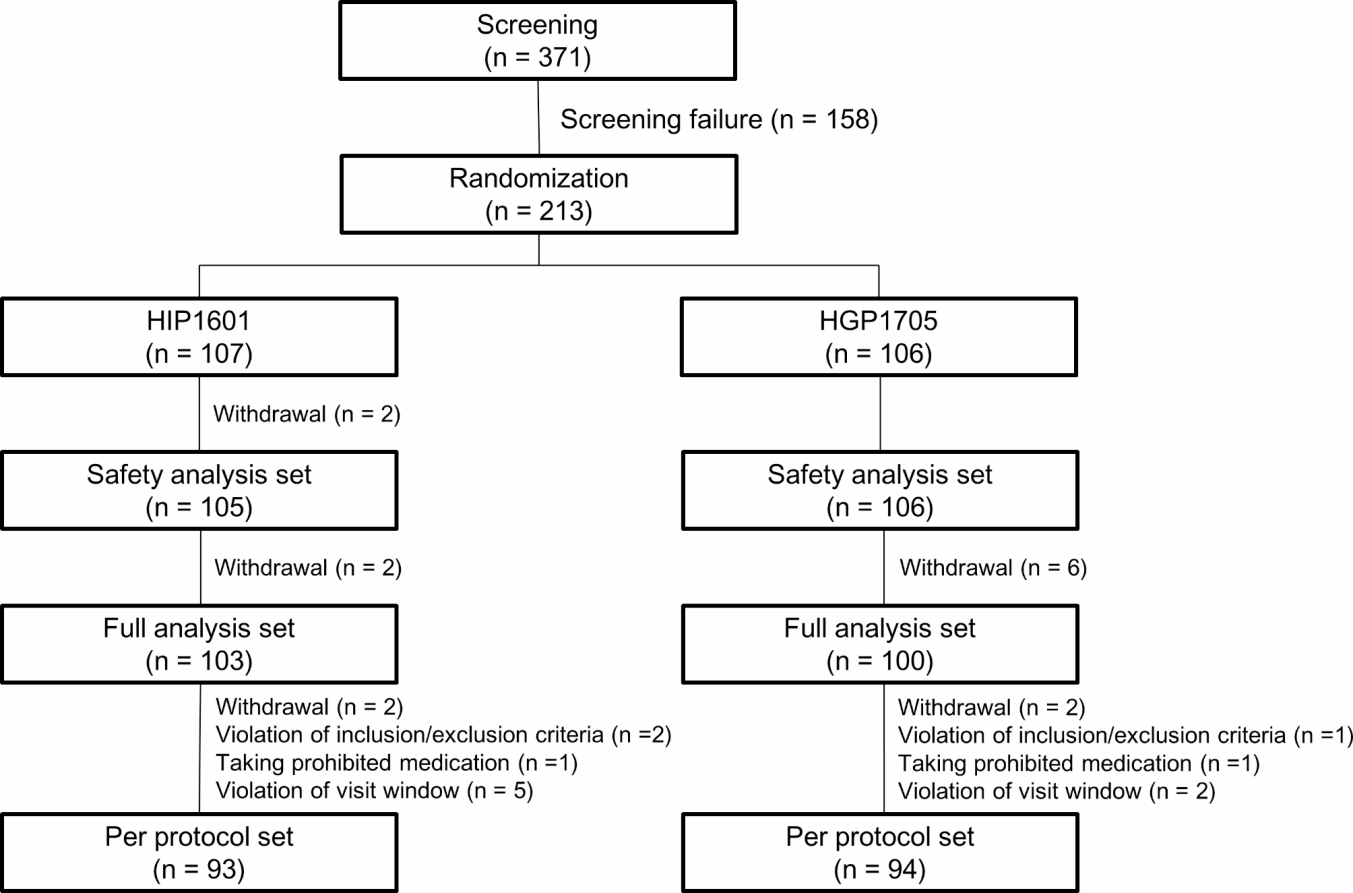
Randomization protocol and patient disposition

Figure 1 shows a schematic representation of the study protocol. An independent staff member created a randomization list using a 1:1 ratio to assign the patients to two groups. Randomization was performed using a stratified block randomization method based on the implementation institute and LA Classification grades (grades A/B and C/D). Eligible patients were randomly allocated to receive either HIP1601 40 mg or HGP1705 40 mg. Both HIP1601 40 mg and HGP1705 40 mg, along with their respective placebos, were administered once daily for 4 or 8 weeks before breakfast. To maintain the blinding of the investigators and patients, the appearance, packing, and labeling of each placebo were identical to those of HIPI1601 (capsule) and HGP1705 (tablet). Antacid (aluminum/magnesium hydroxide; Almagate Tab®; Hanmi Pharma Korea Inc., Seoul, Republic of Korea) tablets were provided as rescue medication for acute intolerable GERD symptoms. All patients were asked to complete a standard questionnaire regarding their medical history and demographic data. They returned to the clinics for the assessment of GERD symptoms, submitted daily symptom records, and returned unused clinical trial drugs at the end of treatment after 4 and 8 weeks. Subsequent endoscopy was performed at week 4 to evaluate the presence and severity of EE. If complete healing of the EE was not confirmed at week 4, additional endoscopy was conducted at week 8. *Helicobacter pylori* screening was performed at the screening visit using either the urea breath test or the rapid urease test. Treatment was completed after 4 weeks if complete healing of the EE was endoscopically confirmed at week 4.

### Efficacy and safety assessment

The primary endpoint of the study was the complete healing rate of EE confirmed endoscopically up to week 8 in the per-protocol set (PPS) population. The complete healing of EE was defined as the LA Classification criterion “Not Present” in endoscopy after administration of the clinical trial drug [9]. The secondary endpoints of the study included the following: (1) complete healing rate of EE confirmed by endoscopy at week 4, (2) complete resolution rate of symptoms at weeks 4 or 8, (3) proportion of symptom-free days and nights after 1, 2, 4, and 8 weeks of treatment, (4) time to sustained resolution of symptoms, and (5) number of rescue medications administered.

Patients were instructed to rate the severity of their worst GERD symptom episodes experienced in the previous 24 h. This information was recorded in a diary every morning using a 4-point scale: 0 (no symptoms), 1 (symptoms with spontaneous remission, not significantly affecting normal activities or sleep), 2 (symptoms with spontaneous but slow remission, somewhat impeding normal activities or sleep), and 3 (symptoms with no spontaneous remission, remarkably impeding normal activities or sleep). Complete resolution of symptoms was defined as patients reporting no symptoms (score 0) during the 7 days before the follow-up visit. Sustained resolution was defined as 7 consecutive days of GERD symptoms recorded as a score of 0 (no symptoms), and the time to sustained resolution of symptoms was determined as the first day of the symptom-free period. To assess the proportion of symptom-free days and nights, the percentage of days without daytime or night-time symptoms during treatment was calculated. Similarly, the percentage of nights without night-time symptoms was assessed using a daily diary.

All safety assessments, including frequency and severity of adverse events, clinical laboratory evaluations, 12-lead electrocardiogram results, vital sign measurements, and physical examination findings, were monitored throughout the study. A TEAE was defined as an adverse event that occurred during treatment and represented a change from baseline. All TEAEs were graded by the investigator as mild, moderate, or severe. Adverse events were considered drug related if they were deemed by the investigator to be possibly related to or related to the study drug. Serious TEAEs were defined as adverse events that could potentially cause death, hospitalization, disability, or life-threatening adverse events.

### Statistical analyses

For estimating the necessary number of subjects, the complete healing rate of EE by week 8 was assumed to be 93.7% in the HGP1705 40 mg group [10–13]. A type I error of 0.025 and a statistical power of 80% were used to demonstrate the non-inferiority of HIP1601 40 mg compared to HGP1705 40 mg. The sample size was calculated to be 89 subjects per group. Assuming a 15% withdrawal rate, a total of 210 subjects were needed for the study, with 105 subjects in each group. Non-inferiority was considered proven if the lower confidence interval of the two-sided 95% confidence interval (CI) of HIP1601 40 mg was above − 10%.

Safety analysis was conducted among those who took at least one dose of the clinical trial drugs after random assignment, for whom data on safety assessments could be obtained. All patients who received at least one dose of investigational drugs and underwent efficacy assessments at least once until the end of the clinical trial were included in the full analysis set (FAS). Among the patients included in the FAS, those who completed the clinical trial in accordance with the protocol were included in the PPS. Patients with major protocol violations or poor compliance (taking less than 70% of the total medications) were excluded from the PPS. Compliance was assessed by counting the unused medications at the completion of 4 or 8 weeks of treatment. For the primary efficacy evaluation, in principle, the PPS was the main analysis set, and the FAS was the supportive analysis set. Secondary efficacy analysis was performed using both PPS and FAS.

The chi-square test or Fisher’s exact test was used to test for associations among various categorical variables, and the independent samples t-test or Wilcoxon’s rank-sum test was used for non-categorical variables. Comparisons between the two groups at weeks 4 or 8 were performed using the Cochran–Mantel–Haenszel test to analyze stratified categorical data (LA Classification, grades A/B and C/D). Statistical analyses were performed using SAS® Version 9.4, SAS Institute, Cary, NC, USA), and a *p*-value < 0.05 was considered statistically significant.

## Results

### Study population and baseline characteristics

In total, 213 patients were enrolled in this clinical trial (Fig. 1). Two patients withdrew before the administration of the clinical trial drug, and 211 patients were included in the safety analysis set. Among them, 203 patients (103 and 100 in the HIP1601 and HGP1705 groups, respectively) were included in the FAS, and 187 patients (93 and 94 in the HIP1601 and HGP1705 groups, respectively) were included in the protocol (14 withdrew, 3 violated the inclusion/exclusion criteria, 2 took prohibited medication, and 7 violated the visit window).

Table 1 summarizes the baseline patient demographics and other characteristics according to the treatment group. Of 203 patients, number of patients with LA Classification grade A, grade B, and grade C EE were 133 (65.5%), 61 (30.1%), and 9 (4.4%), respectively. None of the patients had LA Classification grade D EE. There were no significant differences in any baseline characteristics between the two groups, except for a higher rate of nocturnal dysphagia in the HGP1705 group. The overall compliance with treatment was 97.6%, and there was no statistically significant difference between the two groups (97.2% in HIP1601 group and 98.1% in HGP1705 group, *P* = 0.220).

**Table 1:** Baseline patient demographics and characteristics according to treatment group

	HIP1601 (n = 103)	HGP1705 (n = 100)	*P*-value
Age (mean ± SD), year	52.5 ± 13.5	51.3 ± 13.9	0.499
Male	73 (70.9%)	73 (73.0%)	0.736
Height (mean ± SD), cm	166.8 ± 8.6	166.1 ± 8.5	0.572
Weight (mean ± SD), kg	71.3 ± 12.5	70.3 ± 13.0	0.333
Smoker	21 (20.4%)	23 (23.0%)	0.652
Alcohol	60 (58.3%)	62 (62.0%)	0.586
LA Classification			0.369
Grade A	70 (68.0%)	63 (63.0%)	
Grade B	27 (26.2%)	34 (34.0%)	
Grade C	6 (5.8%)	3 (3.0%)	
Grade D	0 (0%)	0 (0%)	
GERD-related symptoms			
Heartburn	86 (83.5%)	81 (81.0%)	0.585
Acid regurgitation	83 (80.6%)	81 (81.0%)	0.563
Dysphagia	12 (11.7%)	20 (20%)	0.207
Epigastric pain	42 (40.8%)	46 (46.0%)	0.878
Heartburn (night)	42 (40.8%)	47 (47.0%)	0.372
Acid regurgitation (night)	38 (36.9%)	41 (41.0%)	0.549
Dysphagia (night)	3 (2.9%)	11 (11.0%)	0.023
Epigastric pain (night)	24 (23.3%)	21 (21.0%)	0.693
*H. pylori* infection	19 (18.5)	16 (16.0)	0.645
Treatment compliance (mean ± SD)	97.2 ± 5.5	98.1 ± 4.7	0.220
Number of rescue medication administered (mean ± SD)	7.8 ± 8.9	9.3 ± 12.0	0.917

GERD, Gastroesophageal reflux disease; LA, Los Angeles; SD, standard deviation

### Primary endpoint

Based on the PPS analysis by week 8, the complete healing rate of EE in the HIP1601 group was 97.8% (91/93), which was higher than the rate of 96.8% (91/94) in the HGP1705 group (Table 2). The 95% CI on the difference between the two groups ranged from − 4.7 to 7.2. In the FAS analysis by week 8, the complete healing rate of EE (98.1%, 101/103) in the HIP1601 group was higher than that (95.0%, 95/100) in the HGP1705 group, and the 95% confidence interval (CI) of the difference ranged from − 2.5 to 9.7. The lower bounds of the two-sided 95% CI for PPS and FAS analyses were − 4.7% and − 2.5%, respectively. These results exceed the acceptable range of -10% for non-inferiority.

**Table 2:** Healing rate of erosive esophagitis by weeks 8 and 4

	Complete healing of EE	Difference (%) from HGP1705 40 mg	95% CI on the difference	*P*-value^*^
**PPS by week 8**				
HIP1601 (n = 93)	91 (97.8%)	1.0	-4.7, 7.2	< 0.001
HGP1705 (n = 94)	91 (96.8%)			
**FAS by week 8**				
HIP1601 (n = 103)	101 (98.1%)	3.1	-2.5, 9.7	< 0.001
HGP1705 (n = 100)	95 (95.0%)			
**PPS at week 4**				
HIP1601 (n = 93)	85 (91.4%)	-1.2	-8.9, 6.6	0.013
HGP1705 (n = 94)	87 (92.6%)			
**FAS at week 4**				
HIP1601 (n = 103)	95 (92.2%)	3.2	-4.8, 11.3	0.001
HGP1705 (n = 100)	89 (89.0%)			

EE, erosive esophagitis; CI, confidence interval; FAS, full analysis set; PPS, per protocol set

* *P*-value for the non-inferiority test with a margin − 10%

**Fig. 2:**
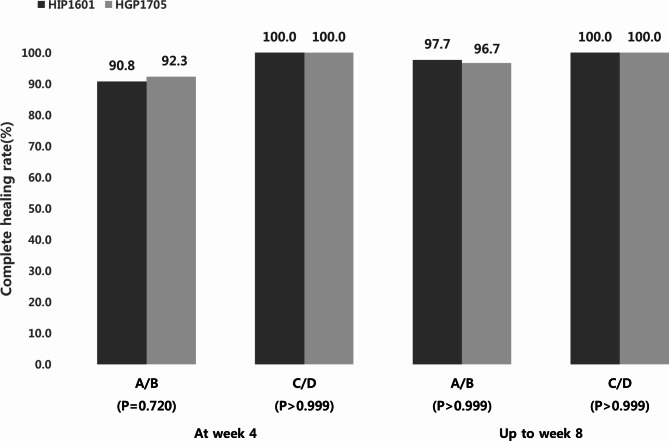
Healing rate of erosive esophagitis at week 4 and up to week 8 according to the Los Angeles Classification (per protocol set)

Furthermore, after 8 weeks of treatment, the complete healing rate of EE was 97.7% for LA Classification grades A/B and 100% for grades C/D in the HIP1601 group. In the HGP1705 group, the rates were 96.7% for grades A/B and 100% for grades C/D. There was no significant difference between both groups (*P* > 0.999) (Fig. 2).

### Secondary endpoints

#### Healing rate of erosive esophagitis at week 4

In PPS analysis at week 4, the complete healing rate of EE in the HIP1601 and HGP1705 groups were 91.4% (85/93) and 92.6% (87/94), respectively, and the 95% CI of the difference between the two groups ranged from − 8.9 to 6.6 (Table 2). Additionally, in the FAS analysis at week 4, the complete healing rate of EE was 92.2% (95/103) in the HIP1601 group and 89.0% (89/100) in the HGP1705 group. The 95% CI of the difference ranged from − 4.8 to 11.3. The lower bound of the two-sided 95% CI for both PPS and FAS analyses exceeded the acceptable range of -10% for non-inferiority.

#### Complete resolution rate of GERD symptoms at week 4 or 8

After 4 weeks of treatment, the complete resolution rate of overall GERD symptoms, including heartburn, acid regurgitation, dysphagia, and epigastric pain, was 49.5% (46/93) in the HIP1601 group and 48.9% (46/94) in the HGP1705 group (Table 3). When considering only the typical GERD symptoms of heartburn and acid regurgitation, the complete resolution rates were 46.7% (28/60) in the HIP1601 group and 57.8% (37/64) in the HGP1705 group. There was no statistically significant difference between the two groups (*P* = 0.214). For each individual symptom, heartburn, acid regurgitation, and epigastric pain showed statistically significant improvements compared to baseline in both groups; however, there were no significant differences between both groups.

**Table 3:** Complete resolution rate of symptoms after treatment (per protocol set)

	HIP1601 (n = 93)			HGP1705 (n = 94)			*P*-value^†^
	Baseline	Week 4	Week 8^*^	Baseline	Week 4	Week 8^*^	
Overall symptoms	92	46 (49.5%)	48 (51.6%)	94	46 (48.9%)	47 (50.0%)	0.941^***^
							0.943^****^
*P*-value^**^		< 0.001	< 0.001		< 0.001	< 0.001	
Heartburn and acid regurgitation	60	28 (46.7%)	30 (50.0%)	64	37 (57.8%)	37 (57.8%)	0.214^***^
							0.383^****^
*P*-value^**^		0.004	0.002		< 0.001	< 0.001	
Heartburn	77	49 (63.6%)	50 (64.9%)	76	55 (72.4%)	55 (72.4%)	0.247^***^
							0.322^****^
*P*-value^**^		< 0.001	< 0.001		< 0.001	< 0.001	
Acid regurgitation	75	51 (68.0%)	52 (69.3%)	77	51 (66.2%)	52 (67.5%)	0.817^***^
							0.811^****^
*P*-value^**^		< 0.001	< 0.001		< 0.001	< 0.001	
Dysphagia	11	4 (36.4%)	4 (36.4%)	18	10 (55.6%)	10 (55.6%)	0.316^***^
							0.316^****^
*P*-value^**^		0.705	0.705		0.467	0.317	
Epigastric pain	36	25 (69.4%)	26 (72.2%)	42	29 (69.0%)	30 (71.4%)	0.970^***^
							0.938^****^
*P*-value^**^		0.011	0.004		0.004	0.002	

The complete resolution rate was defined proportion of patients with ‘score 0’ of severity during the last 7 days prior to the visit

*Cumulative complete resolution rate of symptoms by week 8

**P-value for comparison of before/after administration using McNemar’s test

*** *P*-value for week 4 Using Chi-squre test or Fisher’s exact test

**** *P*-value for week 8 Using Chi-squre test or Fisher’s exact test

The complete resolution rate of GERD symptoms at week 8 was analyzed in 15 patients who did not achieve complete EE healing at week 4. There were no statistically significant differences in any GERD symptoms between the two groups.

#### Proportion of heartburn- and acid regurgitation-free days and nights by week 1, 2, 4, 8 (per protocol set)

**Fig. 3:**
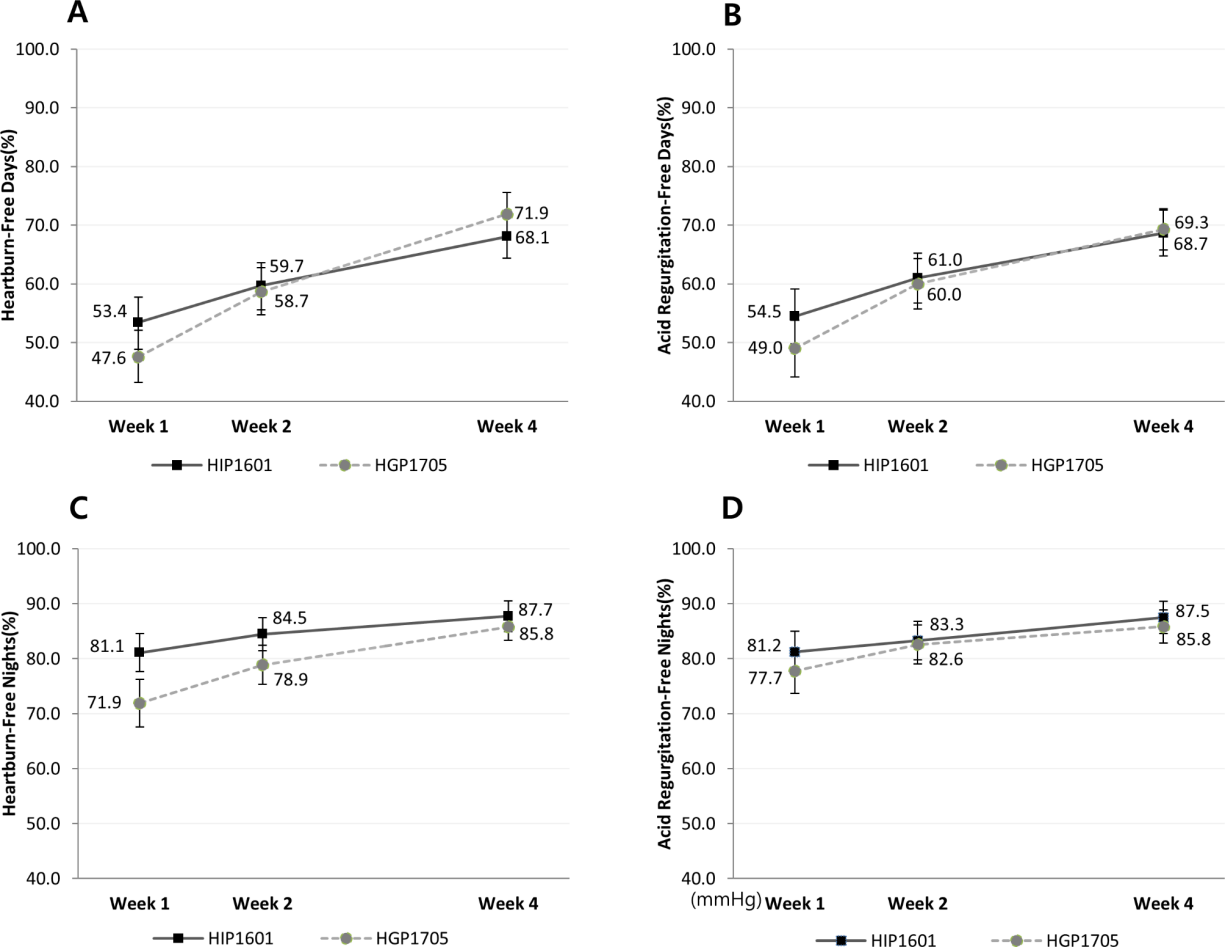
The proportion of symptoms-free days and nights after 1, 2, 4 weeks of treatment (per protocol set). A, the proportion of heartburn-free days after 1, 2, 4 weeks of treatment. B, the proportion of acid regurgitation-free days after 1, 2, 4 weeks of treatment. C, the proportion of heartburn-free nights after 1, 2, 4 weeks of treatment. D, The proportion of acid regurgitation-free nights after 1, 2, 4 weeks of treatment

The proportions of heartburn- and acid regurgitation-free days by week 4 were 68.1% and 68.7% in HIP1601 group, and 71.9% and 69.3% in HGP1705 group, respectively (Fig. 3A and B). The proportion of heartburn- and acid regurgitation-free nights by week 4 was slightly higher in the HIP1601 group compared to that in the HGP1705 group; however, the difference did not reach a clinical significance (87.7% vs. 85.8%, *P* = 0.514; 87.5% vs. 85.8%, *P* = 0.774) (Fig. 3C and D). In both groups, the proportion of heartburn- and acid regurgitation-free days and nights by week 4 was significantly higher than that by weeks 1 and 2.

The proportion of symptom-free days and nights by week 8 was analyzed in 15 patients who did not achieve complete healing of the EE at week 4. There were no statistically significant differences between the two groups.

#### Time to sustained resolution of heartburn and acid regurgitation (per protocol set)

The median time to sustained resolution of heartburn was 7 days (95% CI: 3.0–16.0) in the HIP1601 group and 8 days (95% CI: 5.0–11.0) in the HGP1705 group (Supplementary Table 1). For acid regurgitation, the median time to sustained resolution was 9 days (95% CI: 4.0–15.0) in the HIP1601 group and 8 days (95% CI: 5.0–10.0) in the HGP1705 group. There were no statistically significant differences between the two treatment groups.

The proportion of patients who achieved sustained resolution of nocturnal heartburn and nocturnal acid regurgitation was 87.2% (34/39) and 81.8% (27/33) in the HIP1601 group and 83.3% (35/42) and 86.8% (33/38) in the HGP1705 group, respectively (Supplementary Table 2). The median time to sustained resolution of nocturnal symptoms did not differ significantly between the two groups.

### Number of rescue medication use

The mean number of rescue medications administered in the HIP1601 (7.8 per subject) was lower than that (9.3 per subject) in the HGP1705 group. However, there was no significant difference between the two groups (*P* = 0.917) (Table 1).

### Tolerability and safety

The incidence of TEAEs was 14.3% (15/105; 22 events) in the HIP1601 group and 14.2% (15/106; 20 events) in the HGP1705 group (Table 4). There was no significant difference in the occurrence of TEAEs between both groups. Most TEAEs were rated as mild or moderate in severity, except for one case of tendon rupture, which was considered unrelated to the study drug (HGP1705, 40 mg) by the investigator. No serious adverse events, deaths, or premature study discontinuation due to adverse events were reported.

**Table 4:** Summary of treatment-emergent adverse events (safety analysis set)

	HIP1601 (n = 105)	HGP1705 (n = 106)	Total (n = 211)
Number of subjects with TEAEs^*^	15 (14.3%)	15(14.2%)	30(14.2%)
Intensity			
Mild	13 (12.4%)	13 (12.3%)	26(12.3%)
Moderate	2 (1.9%)	1 (0.9%)	3 (1.4%)
Severe	0 (0.0%)	1 (0.9%)	1 (0.5%)
Relationship			
Yes	2 (1.9%)	6 (5.7%)	8 (3.8%)
No	13 (12.4%)	9 (8.5%)	22 (10.4%)
Number of subjects with serious TEAE	0 (0.0%)	1 (0.9%)	1 (0.5%)
Number of subjects with TEAEs leading to withdrawal	0 (0.0%)	1 (0.9%)	1 (0.5%)

TEAE: treatment-emergent adverse events

*TEAEs: Adverse events with start date on or after administration of the study drug, or pre-existing conditions that worsened during or after study drug administration

## Discussion

The goal of GERD treatment is to alleviate symptoms and prevent complications. Although currently approved delayed-release PPIs have shown benefits in the treatment of GERD, unmet needs remain [14–16]. The HIP1601, a novel dual delayed-release formulation of esomeprazole, has a unique pharmacokinetic profile with two distinct peaks in plasma concentration (1st peak at 1.75 h and 2nd peak at 4.5 h) [8]. This results in extended therapeutic plasma drug concentrations compared to conventional delayed-release PPIs. This randomized controlled study demonstrated that HIP1601 40 mg was highly effective in healing EE and providing relief from GERD symptoms. The safety profile of HIP1601 (40 mg) was acceptable. Furthermore, this study showed that the efficacy of HIP1601 in patients with EE is not inferior to that of HGP1705 (a conventional delayed-release PPI), and the safety of HIP1601 is comparable to that of HGP1705. These results suggest that HIP1601 has the potential to address the unmet needs of GERD treatment and provide an alternative therapeutic option.

Although a new class of drugs, such as potassium-competitive acid blockers (P-CABs), has recently been developed, PPIs remain the first-line drug for GERD treatment [17]. Among PPIs, esomeprazole, the S-isomer of omeprazole, is known to have a slower metabolism, resulting in higher plasma concentrations. Esomeprazole demonstrated better acid control and a higher healing rate of EE compared the other PPIs [18]. In this study, HIP1601 40 mg showed an excellent healing rate of EE, exceeding 90% after 4 or 8 weeks of treatment. Moreover, the healing rate of HIP1601 40 mg by week 8 was superior to that of HGP1705 40 mg, although this difference was not statistically significant. The lower bound of the two-sided 95% CI between HIP1601 40 mg and HGP1705 40 mg was greater than the predefined non-inferiority margin of -10%, meeting the study’s non-inferiority criteria. Therefore, this study demonstrated that HIP1601 40 mg is an effective drug for EE healing, and its efficacy is non-inferior to HGP1705 40 mg.

The spectrum of GERD includes EE, nonerosive reflux disease (NERD), and Barrett’s esophagus. EE is characterized by the presence of erosions in the esophageal mucosa and is commonly graded using the LA Classification system, which has a high level of inter-observer reliability among experienced endoscopists [9]. The severity of EE is closely associated with the extent and duration of gastric acid exposure. Previous studies have shown that patients with more advanced grades of EE (LA Classification grade C or D) tend to have lower symptomatic response and healing rates, as well as a high relapse rate, even with continued treatment using standard-dose PPIs [16]. It has been reported that approximately 4–15% of patients with LA Classification grade C or D EE fail to achieve complete healing after 8 weeks of treatment with standard-dose PPIs [19]. In this study, although the number of patients with LA Classification grade C was small, HIP1601 40 mg demonstrated a high complete healing rate of 100% in this subgroup of patients. This finding suggests that HIP1601 may improve healing rates in patients with more severe forms of EE. However, further studies with larger sample sizes are needed to confirm these results and evaluate the efficacy of HIP1601 in a broader range of patients with different grades of EE.

GERD has a significant impact on the quality of life of affected individuals, as both esophageal and extraesophageal symptoms can be bothersome and affect daily functioning [2, 3]. Heartburn and acid regurgitation are particularly common and troublesome, with a large percentage of patients with GERD considering them the most bothersome symptoms [20]. In previous studies, approximately 73–75% heartburn-free days were observed in patients who received esomeprazole 40 mg for 4 weeks [10, 21, 22]. In the current study, similar improvements in GERD symptoms were observed in both the HIP1601 and HGP1705 treatment groups. After 4 weeks of treatment with HIP1601, the proportions of heartburn- and acid regurgitation-free days was 68.1% and 68.7%, respectively. Although these results were slightly lower than those of previous reports, they were comparable to the findings observed for HGP1705 in this trial. Different statistical methods used to evaluate symptom improvement may have contributed to these differences [10, 21, 22]. In this study, the evaluation of each GERD symptom improvement was specifically conducted only in patients who experienced each respective symptom prior to randomization.

Nocturnal symptoms of GERD can significantly disrupt sleep and reduce the quality of life in patients with GERD. Nocturnal acid breakthrough (NAB), a trop in gastric pH below 4 that lasts for more than 1 h during night, is the main cause of nocturnal symptoms in patients with GERD. NAB presents in a high proportion (75%) of healthy subjects, and a once-daily dose of PPIs, owing to their short half-life, could not eliminate this phenomenon [23]. Various attempts to control NAB have been made; H2 blocker administration at night, split administration of PPIs, and modified-release PPI administration [24, 25]. However, the results of these attempts have been limited. HIP1601 is a dual delayed-release form of esomeprazole that offers an extended duration of plasma drug concentration, and theoretically has the potential to control NAB [8]. In this study, HIP1601 demonstrated high efficacy in controlling nocturnal symptoms of GERD. Although statistical significance was not achieved, the proportion of heartburn- and acid regurgitation-free nights of HIP1601 group was slightly higher than that in HGP1705 group by week 4 (87.7% vs. 85.8%, *P* = 0.514, 87.5% vs. 85.8%, *P* = 0.774). Due to the non-inferiority of the study design and the small number of patients with nocturnal symptoms, it was not possible to determine whether HIP1601 40 mg was superior to HGP1705 40 mg in controlling nocturnal symptoms. Further studies in patients with nocturnal symptoms are required to evaluate the efficacy of HIP1601 in this population.

In addition to effectiveness, the safety of new drugs remains an important issue. Recent reports have highlighted the potential long-term adverse events associated with gastric acid suppression, such as community-acquired pneumonia, clostridium difficile infection, hip fracture, or hypomagnesemia [6]. However, PPIs are well tolerated, and the incidence of adverse events is relatively low when used for short durations. In our study, although the long-term safety of HIP1601 could not be assessed due to the study design, there were no severe adverse events after 4 or 8 weeks of drug administration. The majority of adverse events were mild and moderate, and there were no significant differences compared with conventional PPI (HGP1705).

This study has several limitations. First, most enrolled patients had LA Classification grades A/B EE, with only a small number having LA Classification grades C/D EE. None of the patients had LA grade D EE. Consequently, these findings may not represent the clinical effects in the entire GERD population, particularly in those with more severe EE. Secondly, validated questionnaires specifically designed to assess the severity and improvement of GERD symptoms were not included. However, we analyzed the severity and improvement of GERD symptoms using objective indicators, and the improvement in GERD symptoms was assessed based on dichotomous endpoints (complete resolution or not). Third, the analysis of the complete resolution of GERD symptoms and TEAEs by week 8 was limited to a small subgroup of 15 patients who did not achieve complete healing of the EE at week 4.

## Conclusions

In conclusion, the findings of this study support the efficacy and safety of HIP1601 40 mg as a treatment option for EE healing and alleviating GERD symptoms. The results indicate that HIP1601 is not inferior to HGP1705 40 mg in terms of both efficacy and safety, as demonstrated by comparable healing rates and tolerability profiles. Based on these results, it can be concluded that HIP1601 is effective and well tolerated in patients with GERD.

## CONSORT guidelines

The study adheres to the CONSORT guidelines and a completed CONSORT has been submitted separately.

### Electronic supplementary material

Below is the link to the electronic supplementary material.

Supplementary Material 1

## Data Availability

Relevant data have been presented in the main manuscript. The datasets used and/or analysed during the current study are available from the corresponding author on reasonable request.

## Abbreviations

PPIs: Proton-pump inhibitors
EE: Erosive esophagitis
GERD: Gastroesophageal reflux disease
TEAE: Treatment-emergent adverse events
LA: Los Angeles
PPS: Per-protocol set
CI: Confidence interval
FAS: Full analysis set
P-CABs: Potassium-competitive acid blockers
NERD: Nonerosive reflux disease
