# Supplementary material for: Efficacy and safety of HIP1601 (dual delayed-release esomeprazole) 40 mg in erosive esophagitis compared to HGP1705 (delayed-release esomeprazole) 40 mg: a multicenter, randomized, double-blind, non-inferiority study
Source: BMC Gastroenterol. 2023 Dec 18;23:447. doi: 10.1186/s12876-023-03087-6 (PMC10729464; doi:10.1186/s12876-023-03087-6)
Supplement: Supplementary file 1 — Supplementary Material 1 [file 12876_2023_3087_MOESM1_ESM.docx]

**Supplementary Table 1.** The time to sustained symptom resolution was based on the patient’s diary after treatment.

|  | **HIP1601** | **HGP1705** | ***P*-value*** |
| --- | --- | --- | --- |
| **PPS population** | 93 | 94 |  |
| Heartburn |  |  |  |
| Number of subjects, n | 77 | 76 |  |
| Time to sustained resolution (median), day | 7.0 | 8.0 | 0.331 |
| Acid regurgitation |  |  |  |
| Number of subjects, n | 75 | 77 |  |
| Time to sustained resolution (median), day | 9.0 | 8.0 | 0.573 |
|  |  |  |  |
| **FAS population** | 103 | 100 |  |
| Heartburn |  |  |  |
| Number of subjects, n | 86 | 81 |  |
| Time to sustained resolution (median), day | 9.5 | 8.0 | 0.319 |
| Acid regurgitation |  |  |  |
| Number of subjects, n | 83 | 81 |  |
| Time to sustained resolution (median), day | 9.0 | 9.0 | 0.621 |

* *P*-value for the comparison between treatments were computed using the log-rank test.

**Supplementary Table 2.** The time to sustained resolution of the nocturnal symptoms was based on the patient’s diary after treatment.

|  | **HIP1601** | **HGP1705** | ***P*-value*** |
| --- | --- | --- | --- |
| **PPS population** | 93 | 94 |  |
| Heartburn |  |  |  |
| Number of subjects, n | 39 | 42 |  |
| Sustained resolution (median), day | 5.0 | 3.0 | 0.970 |
| Acid regurgitation |  |  |  |
| Number of subjects, n | 33 | 38 |  |
| Sustained resolution (median), day | 7.0 | 4.0 | 0.227 |
|  |  |  |  |
| **FAS population** | 103 | 100 |  |
| Heartburn |  |  |  |
| Number of subjects, n | 42 | 47 |  |
| Sustained resolution (median), day | 5.0 | 3.0 | 0.751 |
| Acid regurgitation |  |  |  |
| Number of subjects, n | 38 | 41 |  |
| Sustained resolution (median), day | 7.0 | 4.0 | 0.311 |

* *P*-value for the comparison between treatments were computed using the log-rank test.
